# Strawman argument characterises critique of Kamin blocking effect

**DOI:** 10.1177/17470218221104715

**Published:** 2022-06-20

**Authors:** Peter Seraganian

**Affiliations:** Psychology Department, Concordia University, Montreal, Quebec, Canada

**Keywords:** Blocking, replication failure, strawman

## Abstract

This commentary focuses on the robustness of the Kamin blocking effect (KBE) that Maes et al., based upon 15 failures to replicate, have questioned. This challenge to KBE robustness has not gone unaddressed. Soto outlined conceptual as well as methodological issues that cast doubt on the validity of the Maes et al. critique. Despite recognition of certain interpretive issues, Maes et al. have reaffirmed that their failures to replicate are meaningful and call out for further parametric scrutiny of KBE. Faced with such marked differences of opinion with regard to KBE robustness, the present commentary highlights certain methodological features that may have contributed to the Maes et al. failures to replicate: (a) Roles of stimulus salience and independence, (b) Multiple test trials, and (c) Reliance on a single control procedure. It is suggested that the existence of such methodological issues renders it fallacious to argue that the Maes et al. findings represent a true failure to replicate KBE. Instead, the present formulation contends that Maes et al. engaged in a strawman argument in which they have found fault with a distorted version of KBE rather than KBE itself. Although employing variations of paradigms that had earlier been seen to generate KBE, the presence of multiple methodological shortcomings was such as to effectively mask observance of KBE. In affirmation of the Soto’s critique, the replication failure reported by Maes et al. is thus seen as unsurprising. Accordingly, the Maes et al. proposal to remove KBE as a “touchstone for our theories of elementary learning” is argued to be unwarranted. In the face of this protracted dispute, the positing of a strawman argument serves to further cast doubt upon the import of the Maes et al. replication failure, and consequently, to reaffirm KBE as a core phenomenon in the learning domain.

The Kamin blocking effect (KBE), first reported in a series of seminal book chapters (Kamin, 1968, 1969a, 1969b), involves a specific, counter-intuitive finding in which conditioning fails to take place. As depicted in Table 1, rats that had been trained to bar press for food reinforcement on an intermittent schedule received superimposed conditioned stimulus(CS)/shock pairings. KBE consists of a between-group comparison. A control condition (Group NL) received, in Phase 2, eight pairings of an 80 decibel white Noise (N) plus overhead Light (L) compound (NL) trials followed by a brief foot shock. In contrast, the experimental condition (Group N-NL) received identical compound training with the NL compound, but it was preceded, in Phase 1, by 16 trials with the N element alone, paired with foot shock. When presented in the test phase with the L element alone, the group mean suppression ratio on the first test trial for Group N-NL exhibited almost no conditioned fear response to L (i.e., suppression ratio of .45) as opposed to the strong conditioned fear response (i.e., suppression ratio of .04) that was seen in Group NL. Conditioned fear to the L element following NL compound training had been largely *blocked* as a function of prior training in Phase 1 with the N element alone. Thus, simply pairing a CS with a UCS did not appear to be sufficient to generate a conditioned response, but rather, experience prior to the compound trials could proactively affect the strength of a conditioned response.

**Table 1.** Design and group mean test performance for KBE (Kamin, 1969a).

Group	Phase 1	Phase 2	Test	Suppression ratio on test trial with L
Experimental	N+	NL+	L	.45
Control		NL+	L	.05

KBE: Kamin blocking effect.

The “+” represents the delivery of a UCS.

Kamin (1969a) identified a number of factors relevant to the observance of KBE. First, the two stimuli employed in compound training (in his specific case N and L) were independent of each other: acquisition of a conditioned response to one of them did not significantly affect the rate at which conditioned suppression to the other was subsequently acquired. Second, equivalence refers to the similarity in the rate at which a conditioned response was established to each in isolation. Again, in Kamin’s specific CER setting, equivalence was reflected in the fact that about eight CS/UCS pairings of either N or L with shock produced a comparable conditioned emotional response (CER) (i.e., suppression ratio near .05). Finally, it was also pointed out that a certain degree of symmetry was evident in this protocol. More specifically, not only could prior training with N block what was learned about L on subsequent NL compound trials, but also, in different animals, prior training with L could block what was learned about N during subsequent NL compound trials.

Kamin’s (1969a) emphasis upon independence as well as equivalence derived partially from his study of overshadowing. A unique situation arises when two independent stimuli, which are not equivalent in terms of the rate at which they can independently support a conditioned response, are presented as a compound cue. Instead of an 80 decibel white noise (N) as was employed in the basic KBE study depicted in Table 1, a less salient, 50 decibel white noise (N*), was presented in compound (N*L) with the L cue. The N* was independently shown to be capable of supporting a conditioned response when presented alone. However, for rats trained from the outset with the N*L compound, N* elicited almost no conditioned response (i.e., suppression ratio = .45) when presented in isolation on a subsequent test trial. Thus, the more salient L cue could overshadow learning to the less salient N* when the two were presented simultaneously in compound. Accordingly, to obviate the imbalance that such overshadowing could generate, equivalence and independence were emphasised as factors that could markedly affect the degree to which for KBE took place. Although the roles of salience, independence, and symmetry, which were all empirically documented in Kamin’s initial reports, may not, necessarily, be fully reflected in the numerous settings in which KBE has subsequently been observed (Bouton & Hall, 2019; Mackintosh, 1976), the contribution of such factors would still seem to be relevant with respect to the exploration of KBE robustness.

Following publication of Kamin’s (1968, 1969a, 1969b) book chapters, reports soon emerged (see Seraganian, 1974, p. 226) confirming KBE: (a) in a variety of aversive and appetitive paradigms, (b) with different species, and (c) with diverse responses. Given such robustness, KBE emerged as a cornerstone in contemporary learning theory (Bouton & Hall, 2019; Mackintosh, 1974). However, after more than a half-century of largely supportive findings, the Maes et al. (2016) 15 failures to replicate stands out as somewhat of an anomaly.

Appreciable methodological diversity is seen in the multiple failures to replicate reported by Maes et al. (2016). More specifically, the experiments exhibited the following: (a) both aversive and appetitive conditioning settings, (b) a variety of conditioned behaviours including lever-pressing for either food pellets or sucrose solution, nose poking for food or simple food magazine entry, and (c) both mice or rats served as subjects. In contrast to such methodological diversity, the overall experimental design, as depicted in Table 2, was largely uniform across the 15 experiments. For Experimental group, Phase 1 training with Stimulus A was followed in Phase 2 by training with the AX compound. Thus, the training received by Experimental group mimicked to some degree that employed in Kamin’s (1968) experimental condition (i.e., Group N-NL). However, in terms of training given to Control group, a key methodological difference from Kamin (1968) is evident. As characterised in Table 2, for Control group, prior to training with compound AX cues, training was given with Stimulus B.

**Table 2.** General design of Maes et al. (2016), 15 experiments with group mean results in the test phase for first 3 experiments.

Group	Phase 1	Phase 2	Test	Mean suppression ratio in test in experiments 1, 2, and 3		
				1	2	3
Experimental	A+	AX+	X	.02	.54	.54
Control	B+	AX+	X	.02	.50	.56

The “+” represents the delivery of a UCS: A, B, and X represent different auditory and visual conditioned stimuli presented in a steady or pulsed manner.

On the basis of systematic analyses by Taylor et al. (2008), Maes et al. (2016) affirmed that, among a number of alternatives, the control protocol that was preferable involved the acquisition of a conditioned response in Phase 1 to Stimulus B prior to AX compound training in Phase 2. Clearly, there are grounds for exploring in greater depth what specifically it was about Phase 1 training that contributes to KBE. How one deals with this issue directly affects choices as to what constitutes an appropriate control condition. For instance, could Phase 1 exposure to a UCS alone or random CS/UCS presentations affect what was learned with compound cues in Phase 2?

Admittedly, there exists a certain degree of procedural similarity in the overall designs as presented in Tables 1 and 2. However, the proposition to be developed here is that the correspondence across design is nominal and masks critical methodological disparity. Adapted from Appendix F in Maes et al. (2016), Table 3, which sets out stimuli employed across the 15 experiments, anchors the repeated reference made below to various attributes of these stimuli.

**Table 3.** Stimuli (A, B, and X) employed by Maes et al. (2016) in the 15 experiments.

Experiment	A/B	X
1	Flashing light/steady light	Pulsing 3.500 Hz tone
2	1.000 Hz-tone/pulsing tone (1.500 and 2.500 Hz)	Flashing light
3	1.000 Hz tone/pulsing tone(1.500 and 2.500 Hz)	Flashing light
4	Flashing light/steady light	Tone
5–6	300 Hz tone/clicker	White noise
7–10	1.000 Hz tone/3.000 Hz tone	Clicker
11	Clicker/white noise/1.000 Hz tone	
12	Pulsing 1.000 Hz/pulsing 7.000 Hz tone	Clicker
13	Pulsing 1.000 Hz/pulsing 7.000 Hz tone	Clicker
14	1.000 Hz tone/clicker	White noise
15	1.000 Hz tone/white noise	Bright light

Soto (2018) has described two prominent features that contributed to the failures to replicate KBE. First, in 10 experiments, namely 5–14, the fact that Stimuli A and X were from the same modality resulted in generalisation from A to X that detracted from the observance of KBE. Second, in three other experiments (specifically 2, 3, and 15), a floor effect in Control group (e.g., suppression ratio during test phase near .50) meant there was no conditioned response that could be blocked by subjects in Experimental group. The recognition originally by Maes et al. (2016) of such a floor effect in three experiments led to a reanalysis of their data excluding these three. When this reanalysis, based on only 12 experiments, still yielded negative results, the inference was drawn that the non-significant group difference in all 15 experiments collectively undermined the robustness of KBE. Even though, in some instances, floor and ceiling effects can be corrected with statistical techniques (Liu & Wang, 2021), the rationale for such an approach, in this case, is unclear. Accordingly, Soto (2018), based both on the generalisation problem in 10 experiments and on the questionable interpretation in three experiments with floor effects, affirmed that the Maes et al. (2016) critique of the validity of KBE was “far-fetched.”

In a lengthy rebuttal, Maes et al. (2018) sought to counter Soto’s criticisms and thereby to reaffirm that their failures were meaningful. First, it was argued that “there are no logical or empirical reasons to conclude that the use of same-modality stimuli should reduce the chances of finding blocking by increasing generalization.” In support of this contention they cite Robinson et al. (2017), who reported that generalisation, in rats, between a pair of auditory stimuli, specifically from a clicker to a tone, was “restricted” when the stimuli differed in terms of novelty, as is typically the case in blocking experiments. However, closer examination of the Robinson et al. (2017) findings reveals that although generalisation may have indeed been “restricted,” there was still appreciable generalisation from clicker to tone, just less generalisation than in their non-novel condition. Adapting the Maes et al. (2016) nomenclature, such a generalisation decrement from Stimulus A to X, even if it were to some degree “restricted,” could yet be sufficient to disrupt KBE. Consequently, in 10 of the 15 experiments (namely Experiments 5–14) reported by Maes et al. (2016) as KBE failures, the lack of independence between Stimulus A and X, and the resulting generalisation decrement could still have contributed to the negative results. Thus, a precondition identified by Kamin (1968), namely, stimulus independence, or more specifically the lack there of, may still stand as a contributing factor underlying the Maes et al. (2016) multiple failures to replicate.

Second, the potential impact of stimulus salience, although nominally recognised, was argued to be ambiguous. More specifically, following acknowledgement that differential stimulus salience could have contributed to the “floor” effect, seen in Experiments 2, 3, and 15, Maes et al. (2018) persisted in including the results from these three experiments as exemplars of KBE failure.

Thus, disagreement with regard to KBE robustness is readily apparent in the current literature. Admittedly, as outlined by Maes et al. (2016) in Appendices A–E, versions of the diverse methodologies used in their 15 experiments have, at times, previously yielded positive results with respect to KBE. However, the argument to be developed here is that specific suboptimal methodological choices, fundamental to the Maes et al. (2016) experiments, have yielded data that could have readily masked earlier observance of KBE. Three of these problematic procedural details are delineated below:

(a) Roles of stimulus salience and independence,
(b) Multiple versus single test trial,
(c) Reliance on a single control procedure.

## Roles of stimulus salience and independence

Marked differences in the salience of stimuli employed in the initial three experiments can be identified. In Experiment 1, which was run in the dark, with no background house light illumination, Stimulus X was a pulsing tone with Stimuli A and B the flashing or steady illumination of the house light. For Experiments 2 and 3, there was a background house light on throughout the session unless it was flashing to serve as Stimulus X. Stimuli A and B were a steady or pulsing tone. In Table 2, on the right-hand side, the suppression ratios on test trials for Experimental and Control groups in the first three experiments are presented (exact values were extrapolated from Figure 2 in Maes et al., 2016). There was a strong conditioned response (i.e., suppression ratios approaching .00) for both groups in Experiment 1 with no virtually no conditioned response (i.e., suppression ratios around .50) for both groups in Experiments 2 and 3. This absence, in all three experiments, of any group difference in test performance was interpreted as evidence for a failure to replicate KBE.

An alternate explanation for these findings can be developed. In Experiment 1, the pulsed tonal cue, Stimulus X, essentially overshadowed the visual cues, either a steady or flashing light, and consequently, yielded strong responding in the test phase. In Experiments 2 and 3, the steady or pulsed tonal cues, Stimuli A and B, overshadowed the flashing house light, Stimulus X, when it was presented in Phase 2 compound training and resulted in virtually no responding in the test phase to the light in both groups. Overshadowing is, in part, a reflection of the differential salience of the two cues that comprise the compound (Mackintosh, 1976). Given these circumstances, the claim of failure to replicate KBE, in these three specific instances, seems unfounded. Moreover, in Experiments 2 and 3, given test suppression ratios around .50, how can one argue for KBE failure for those in Experimental group, if, to begin with, those in Control group exhibit virtually no conditioned response that can be blocked? The position taken here is that in these three experiments, failures were more a consequence of differential stimulus salience as opposed to any shortcomings with respect to KBE itself. In essence, not only did Maes et al. (2016) ignore the salience precondition described by Kamin (1969a), but also the marked differential salience of stimuli may be the underpinning for some of their negative findings.

The second precondition identified by Kamin (1969a), namely stimulus independence, also encounters difficulty. Soto (2018) identified 10 of the Maes et al. (2016) experiments, namely 5–14, in which Stimuli A and X were from the same modality and thus unlikely to be independent of each other. Admittedly, there have been reports (Seraganian, 1974) in which stimulus modality did not influence KBE. In an operant discrimination paradigm, pigeons given discrimination training with either line-orientation or auditory cues in isolation had these cues paired with colour cues in compound training. A subsequent test with the colour cues alone indicated that both pretraining conditions with stimuli from the same (i.e., visual) or a differing (i.e., auditory) modality yielded KBE. Thus, modality does not necessarily affect the extent to which KBE is observed.

The question arises, nevertheless, to what degree did modality confounds in Maes et al. (2016) experiments compromise the independence of Stimuli A and X. In particular, concerns with stimulus independence extend beyond issues of traditional modality such as visual or auditory. In the majority of the 15 studies interpreted by Maes et al. (2016) as KBE replication failures stimuli, both visual and auditory, could sytematically differ in their pattern of presentation, with cues presented either in a continuous, or in a pulsed, interrupted (i.e., on and off) fashion.

Although not stated explicitly, an apparent assumption appears to have been that, beyond what is recognised with traditional stimulus modalities, such patterns of stimulus presentation have little impact on generalisation. There are data (Seraganian & Popova, 1976) that refute such a premise. Dogs that had first learned a paw flexion response for food, then learned to discriminate a pulsed from an uninterrupted tone. In a subsequent test phase, the immediate transfer of this auditory discrimination to visual stimuli (i.e., pulsed light vs uninterrupted light) was observed. More specifically, a strong flexion response was seen on the first presentation of a pulsed light, whereas no response to the uninterrupted light was seen. Thus, the transfer of discriminative control is not limited to conventional stimulus modality (e.g., auditory or visual), but can occur also across modality, depending on the pattern (i.e., pulsed vs uninterrupted) in which stimuli are presented.

In the Maes et al. (2016) experiments, possible instances of comparable, cross-modal transfer can be identified. Take, e.g., Experiment 1 in which Stimuli A and B were a flashing light and a steady light, counterbalanced; Stimulus X was a pulsing tone. For half of the animals in Experimental group, generalisation based on pattern (i.e., the intermittency of the stimulus) from the flashing light (A) to the pulsing tone (X) could act to facilitate rather than block responding in the test to Stimulus X. Even though, through counterbalancing, this disruptive effect might only be present in half of the subjects, the situation is clearly far from optimal in terms of the detection of KBE. Similar cross-modal A to X transfer may also be at play in Experiments 2 and 3 (in these instances from pulsing tone to flashing light). In summary, multiple cases of cross-modal transfer based on stimulus pattern leaves open the prospect of recurrent confounding factors in the Maes et al. (2016) experiments. Such ambiguity with regard to the role of stimulus independence further weakens the argument as to the apparent lack of KBE robustness.

## Multiple versus single test trial

In the first three experiments reported by Maes et al. (2016), the test phase consisted of the average performance across four presentations of Stimulus X. Other than the assertion that this was a protocol utilised previously in the literature, little rationale is provided for averaging test performance across multiple extinction trials. In fact, over their 15 separate studies, Maes et al. (2016) employed up to 10 presentations of Stimulus X in the test phase. In contrast to this repeated use of multiple test trials, in Kamin’s (1968) research, only one test trial was employed. Although data for individual trials were archived by Maes et al. (2016), at Open Science Framework (https://osf.io/fcwnr/view_only_754693fa2907497a9ad8013a63813781), their analysis consistently focused on test performance averaged across multiple test trials.

The rationale for Kamin’s choice of a one-trial test was clear. Performance on the first test trial largely reflects, retroactively, the impact of training experienced earlier in Phases 1 and 2. After the first test trial has taken place, and for any subsequent test trials, given that the test phase is conducted with no further presentation of the UCS (i.e., in extinction), complications arise. More specifically, the proactive effect accruing due to the extinction of the conditioned response would now come into play. Given that such extinction is taking place for both Experimental and Control groups, the response strength in the test phase is likely to not only decline over multiple test trials but also to converge across the two groups. Thus, it is probable that multiple extinction test trials would serve to shrink any group difference that was present on the initial test trial. Accordingly, the use of multiple as opposed to a single trial in the test phase would seem to be another methodological feature that could serve to compromise detection of KBE.

## Reliance on a single control procedure

A third difficulty arises with respect to the precise training given to Control group. Maes et al. (2016) contend that the Taylor et al. (2008) analysis argued strongly for the use of a control protocol in which exposure, in Phase 1 to conditioned stimuli, independent of those employed in Phase 2 compound training, was carried out. In Table 2, this Phase 1 experience for the Control group is represented as B+. With reference to Taylor et al. (2008), Maes et al. (2016) categorically assert: “In conclusion, the control procedure used in the current experiments is to be regarded as the most appropriate of the control groups commonly used in blocking experiments” (p. 51). Nevertheless, closer examination of Taylor et al. (2008) with regard to what constitutes an optimal control procedure brings to light a somewhat different scenario. In fact, Taylor et al. (2008) offer a more sanguine view of the adequacy of the B+ control protocol. The KBE literature exhibits a number of other viable options for use in Control group conditions (e.g., UCS alone, backward UCS/CS pairings, random CS/UCS presentations). In the face of such credible alternatives, coupled with the spectre of compromised stimulus independence B to X, which Mackintosh et al. (1980) noted as a concern, the sole reliance on the B+ control, particularly when it comes to KBE robustness, can be questioned.

Thus, three critical aspects of the Maes et al. (2016) methodology, namely stimulus features, test phase length, as well as the control protocol, all appear to be suboptimal with respect to replication of KBE. Indeed, relative to the methodology employed in Kamin’s (1968) original and much subsequent KBE experimentation, multiple, questionable methodological choices seemed to have prevailed. The preponderance of such critical methodology choices, characteristic of the Maes et al. (2016) multiple failures to replicate, raises the prospect of a *strawman* argument.

Itamar Shatz (n.d.) at the Effectiviology website has defined a strawman as a fallacious argument that distorts a particular stance to make it easier to discredit. Essentially, the person using the strawman professes to criticise a particular position. However, what is actually being criticised is a distorted, only superficially similar, version of that position. In all likelihood, the object of the criticism, in this case Kamin, wouldn’t recognise the stance taken as valid. This commentary has elaborated in some depth on major discrepancies between the methodologies employed in Kamin’s (1968) initial report of KBE to that employed in the Maes et al. (2016) multiple replication failures. It is proposed that the specious concordance in experimental methodology masks a fallacy: the Maes et al. (2016) claims of KBE replication failures are the fruit of a strawman argument.

Admittedly, with systematic as opposed to direct replication, some degree of methodological variation is to be expected (Sidman, 1960). However, at some point, methodological disparity, if excessive, can be the underpinnings for failure to replicate. In this context, what bears closer scrutiny is the repeated assertion by Maes et al. (2016) that their protocols largely mimicked methodologies that had, in prior research, successfully replicated KBE. In Maes et al. (2016) Appendix A, stimuli in Experiments 1–4 are asserted to be similar to those employed by Mackintosh et al. (1980) in which Stimuli A and B consisted, respectively, of a diffuse, overhead 60 W light and a flashing 3 W light emanating from a small jewel lamp. Directed behaviour in rats, associated with small, localised, visual cues, can generate “sign-tracking” (Crowell & Bernhardt, 1979) that can markedly affect the strength of a conditioned response or discrimination. The Maes et al. (2016) use of a house light as opposed to the small, localised stimuli employed by Mackintosh et al. (1980) may have attenuated the enhanced conditioning which is often seen in settings in which “sign-tracking” takes place. Consequently, the weaker Phase 1 conditioned response, which the Maes et al. (2016) protocol likely generated, may have contributed to their failure to observe KBE. At the least, the visual stimuli employed in the two studies differed considerably. Moreover, Mackintosh et al. (1980) actually acknowledged (p. 390) that generalisation between visual cues in their Control group, an issue that plagues a number of the Maes et al. (2016) experiments, may have affected the degree to which blocking took place.

The principal thrust of this commentary is to support Soto’s (2018) critique and thus to reaffirm that the Maes et al. (2016) failures to replicate KBE are neither “surprising nor informative.” The strawman argument takes this dispute a step further. In comparison with research that has systematically replicated KBE, it is argued that the Maes et al. (2016) failures are characterised by the distortion of key methodological elements with respect to stimulus characteristics, test duration, as well as the control condition. Such overriding methodological deformation undermines the credibility of the Maes et al. (2016) contention that their “failures raise doubts regarding the canonical nature of the blocking effect and call for a reevaluation of the central status of blocking in theories of learning.”

In part, what remains to be determined is a choice between opposing interpretations of the Maes et al. (2016) findings. Do these failures to replicate truly compromise KBE robustness, or instead, are the multiple failures to replicate merely the product of a flawed, strawman argument? The resolution of this issue should then help to resolve whether KBE remains as a core phenomenon in our understanding of the conditioning process, or whether, as a consequence of its compromised robustness, declines notably in significance.

Careful exploration of the necessary and sufficient conditions associated with core learning phenomenon is fundamental to the discipline. The position taken here is that the original Kamin (1968, 1969a, 1969b) studies set a high bar for the establishment of KBE as such a core conditioning phenomenon: thoughtful, critical analysis accompanied Kamin’s original analysis of the role of multiple factors in KBE. In contrast, the Maes et al. (2016) failures to replicate stand as a relatively low bar with respect to what constitutes legitimate replication failure. Not only did they engage in a strawman argument but also their overall interpretive credibility is, at times, suspect. Indisputably, any phenomenon fundamental to our understanding of the learning process should, at all times, be subject to rigorous experimental scrutiny. However, as Mackintosh (1997) so forcefully argued, lax experimental scrutiny could eventually relegate conditioning theory to the “scrap heap.” If a strawman argument can be legitimised, and subsequently allowed to undermine the status of an established phenomenon, one may be hastening such an uncertain development.

## References

[bibr1-17470218221104715] Bouton M. E. Hall G. (2019). Learning theory. In Dunn D. S. (Ed.), Oxford biographies in psychology. Oxford University Press.

[bibr2-17470218221104715] Crowell C. R. Bernhardt T. P. (1979). The feature-positive effect and sign-tracking behavior during discrimination learning in the rat. Animal Learning & Behavior, 7(3), 313–317.

[bibr3-17470218221104715] Kamin L. J. (1968). “Attention-like” processes in classical conditioning. In Jones M. R. (Ed.), Miami symposium on the prediction of behavior (pp. 9–31). University of Miami Press.

[bibr4-17470218221104715] Kamin L. J. (1969a). Predictability, surprise, attention, and conditioning. In Campbell B. A. Church R. M. (Eds.), Punishment and aversive behavior (pp. 279–296). Appleton-Century-Crofts.

[bibr5-17470218221104715] Kamin L. J. (1969b). Selective association and conditioning. In Mackintosh N. J. Honig W. K. (Eds.), Fundamental issues in associative learning (pp. 42–64). Dalhousie University Press.

[bibr6-17470218221104715] Liu Q. Wang L. (2021). T-Test and ANOVA for data with ceiling and/or floor effects. Behavioral Research Methods, 53(1), 264–277. 10.3758/s13428-020-01407-2 32671580

[bibr7-17470218221104715] Mackintosh N. J. (1974). The psychology of animal learning. Academic Press.

[bibr8-17470218221104715] Mackintosh N. J. (1976). Overshadowing and stimulus intensity. Animal Learning & Behavior, 4(2), 186–192. 964444 10.3758/bf03214033

[bibr9-17470218221104715] Mackintosh N. J. (1997). Has the wheel turned full circle? Fifty years of learning theory, 1946-1996. Quarterly Journal of Experimental Psychology, 50, 879–898.

[bibr10-17470218221104715] Mackintosh N. J. Dickinson A. Cotton M. M. (1980). Surprise and blocking: Effects of the number of compound trials. Animal Learning & Behavior, 8, 387–391.

[bibr11-17470218221104715] Maes E. Boddez Y. Alfei J. M. Krypotos A.-M. D’Hooge R. De Houwer J. Beckers T. (2016). The elusive nature of the blocking effect: 15 failures to replicate. Journal of Experimental Psychology: General, 145(9), e49–e71. 10.1037/xge0000200 27428670

[bibr12-17470218221104715] Maes E. Krypotos A.-M. Boddez Y. Alfei Palloni J. M. D’Hooge R. De Houwer J. Beckers T. (2018). Failures to replicate blocking are surprising and informative—Reply to Soto (2018). Journal of Experimental Psychology: General, 147(4), 603–610. 29698031 10.1037/xge0000413 PMC5935240

[bibr13-17470218221104715] Robinson J. Whitt E. J. Jones P. M. (2017). Familiarity-based stimulus generalization of conditioned suppression. Journal of Experimental Psychology: Animal Learning and Cognition, 43(2), 159–170. 28383938 10.1037/xan0000134 PMC5410874

[bibr14-17470218221104715] Seraganian P. (1974). A within and between modality analysis of blocking. Canadian Journal of Psychology, 28, 225–238. 4426010 10.1037/h0081989

[bibr15-17470218221104715] Seraganian P. Popova Y. I. (1976). Cross modal transfer of a conditioned flexion response in dogs. Pavlovian Journal of Biological Science, 11, 162–174. 934719 10.1007/BF03000293

[bibr16-17470218221104715] Shatz I. (n.d.). Strawman arguments: What they are and how to counter them. https://effectiviology.com/straw-man-arguments

[bibr17-17470218221104715] Sidman M. (1960). Tactics of scientific research: Evaluating experimental data in psychology. Basic Books. 10.1007/BF03392531 PMC2733433 22478062

[bibr18-17470218221104715] Soto F. A. (2018). Contemporary associative learning theory predicts failures to obtain blocking: Comment on Maes et al. (2016). Journal of Experimental Psychology: General, 147(4), 597–602. 29698030 10.1037/xge0000341

[bibr19-17470218221104715] Taylor K. M. Joseph V. T. Balsam P. D. Bitterman M. E. (2008). Target-absent controls in blocking experiments with rats. Learning & Behavior, 36, 145–148. 18543714 10.3758/lb.36.2.145 PMC4864974

